# Hemorrhoidectomy for elderly patients aged 75 years or more, before and after studies

**DOI:** 10.1016/j.amsu.2020.04.045

**Published:** 2020-05-16

**Authors:** Masateru Yamamoto, Masanobu Ikeda, Tomio Matsumoto, Masahiko Takemoto, Ryo Sumimoto, Tsuyoshi Kobayashi, Hideki Ohdan

**Affiliations:** a Department of Surgery, National Hospital Organization Yanai Medical Center, Yamaguchi, Japan; b Department of Gastroenterological and Transplant Surgery, Graduate School of Biomedical and Health Sciences, Hiroshima University, Hiroshima, Japan

**Keywords:** Benign anorectal disease, Elderly, Hemorrhoid, Hemorrhoidectomy, Postoperative complications

## Abstract

**Background:**

The incidence of hemorrhoids requiring hemorrhoidectomy among the elderly has been increasing. Old age is sometimes considered a contraindication for surgery. The relationship between age and complications of hemorrhoidectomy for elderly patients is not well established. This study aimed to compare the clinicopathological features and postoperative outcomes of hemorrhoidectomy in the elderly (≥75 years old) and non-elderly patients (<75 years old).

**Methods:**

A total of 100 patients who underwent hemorrhoidectomy for hemorrhoids of Goligher classification grades 3 and 4 at our institution between 2014 and 2018 were enrolled. The clinical characteristics were compared between the elderly and non-elderly patients. Pain scores were measured at 6, 12, 24, and 48 h after surgery. The risk factors for postoperative complications were identified.

**Results:**

A total of 34 patients were classified as elderly patients. In the elderly group, aspartate aminotransferase levels were higher while the albumin levels and cholinesterase levels were lower and the platelet counts were significantly lower. The blood urea nitrogen levels were higher and estimated glomerular filtration rates and hemoglobin levels were significantly lower in the elderly group. The pain scores significantly decreased at 48 h postoperatively compared to those recorded at 6 h postoperatively in both groups. Multivariate analysis identified Goligher classification grade 4 and high neutrophil to lymphocyte ratio at the indicators of complications.

**Conclusions:**

Hemorrhoids due to impairment of liver function and kidney function were dominant in elderly patients. Aging itself was not a risk factor for postoperative complications.

## Highlights

• The feasibility of hemorrhoidectomy for the elderly is not remained clear.
• The incidence of postoperative complications was comparable regardless of age.
• The risk factor for complication was Goligher classification grade 4 and high NLR.

## 1 Introduction

Hemorrhoids are one of the most common diseases in both men and women, based on screening colonoscopy data; about 38% of the population has hemorrhoids, and only 44% of those with hemorrhoids on colonoscopy reported symptoms [1].

Hemorrhoids are graded according to the grade of prolapse, where, in patients with III and IV degrees, hemorrhoidectomy is required [2]. Surgery is one of the main therapeutic strategies for providing timely treatment, particularly for patients for whom conventional methods have failed, leading to the development of the complications.

The increasing aging population is a worldwide issue undergoing a significant demographic trend toward aging of its population, owing to decreased birth rates and increased longevity [3]. In Japan, the elderly population (https://www.stat.go.jp/english/data/jinsui/2018np/index.html) over the age of 65 has consistently increased since 1950, with an estimate of 35.57 million in 2018, accounting for 28.1% of the total population. The population over the age of 75 accounts for 17.96 million (14.2%). Japan has one of the greatest longevity populations in the world and one of the most advanced aging societies.

The number of surgical procedures in the elderly population has increased in the past few decades [4,5]. Older patients undergoing surgery are at increased risk of mortality and morbidity [6,7], because older patients are more likely to have clinically significant comorbidities, malnutrition, and impairment of reserve function than younger patients [8]. However, recent advances in surgical techniques and perioperative management have reduced the postoperative morbidity and mortality in patients with surgery [4,[9], [10], [11], [12], [13]]. Although the presence of hemorrhoids is not a life-threatening condition, diagnosis and management of hemorrhoids may be challenging, especially in the elderly [14].

Therefore, this study aimed to analyze the clinicopathological features, perioperative outcomes, and risk factors for adverse events in elderly patients with hemorrhoids in comparison with those of the younger patients. To the best of our knowledge, this study is the first study to determine the safety and efficacy of hemorrhoidectomy for elderly patients with hemorrhoids.

## 2 Methods

### 2.1 Patient selection

A total of 100 who underwent hemorrhoidectomy for hemorrhoids with Goligher classification grades 3 and 4 [15] at our institution between January 2014 and December 2018 were included in the present study. In Japan, elderly people aged ≥75 years are defined as the elderly in the latter stage of life from the viewpoints of their frailty and their medical costs (https://www.mhlw.go.jp/english/wp/wp-hw3/02.html), thus we defined patients aged ≥75 years as the elderly. Hemorrhoids were identified by anoscope and evaluated for degree of mucosal elevation of rectal columns with Goligher classification [15]. Patients with previous hemorrhoid surgeries, intestinal bowel disease or colorectal carcinoma were excluded. Prior to the operation, blood samples were collected. C-reactive protein to albumin ratio (CAR) was calculated as the patient's serum CRP level (mg/dL) divided by the serum albumin level (g/dL). The neutrophil to lymphocyte ratio (NLR) was calculated as neutrophil counts divided by lymphocyte counts. The lymphocyte to monocyte ratio (LMR) was calculated as lymphocyte counts divided by monocyte counts.

The baseline clinicopathological findings were retrieved and reviewed from the hospital database. This study was the approved by the Institutional Review Board (Provided ID Number: Y-1-11) on the basis of the Ethical Guidelines for Clinical Research of the Ministry of Health, Labour and Welfare in Japan. Informed consent was obtained according to the Declaration of Helsinki. The work has been reported in line with the STROCSS criteria [16].

### 2.2 Surgical technique

Under lumbar subarachnoid anesthesia, set to the jackknife position, to relieve postoperative pain, we first pull the rectal sphincter up in various directions vertically and horizontally with a stalk anoscope, and expand it. Epinephrine injection 200 × 10^3^ dilution was given subcutaneously to reduce intraoperative bleeding. Ligation and Excision is performed according to the modified version of the Milligan-Morgan procedure [17]. To prevent stenosis after resection, the width of the skin incision should be approximately 50% of the seize of the hemorrhoid at the widest point. After skin incision, the flap with hemorrhoidal tissue is removed while keeping the internal anal sphincter intact by using electrocautery device. The upper rectal artery is punctured and ligated, and the flap with the hemorrhoid is excised. The anal epithelium is closed by suture from the center and the periphery is opened by a semi-closed method. If there are three or more hemorrhoidal piles, the accessory hemorrhoids are ligated alone to prevent postoperative stenosis.

### 2.3 Postoperative management

After surgery, patients are managed according to the clinical path. Patients are administered NSAIDs three times daily and received additional analgesics when pain is severe. Postoperative pain was assessed at 6, 12, 24, and 48 h using a pain score scale, as follows: 0 = no pain, 1 = mild, 2 = moderate, and 3 = severe pain. They were discharged 2 days after surgery. Patients were assessed by a surgeon in the hospital out-patient clinic at 1 week after the surgery and then at 1, 2, 6, and 12 months, and finally at 2 years after surgery.

Regarding complications, postoperative bleeding occurs mostly from the arteries or veins in the root. Recurrence was defined excluding skin tags or thrombosed hemorrhoids. Rectal stenosis was defined as the loss of compliant natural elasticity of the anal orifice, followed by an abnormal stiffness and fibrosis.

### 2.4 Statistical analysis

All statistical analyses were performed using JMP 14 software (SAS Institute Inc., Cary, NC, USA). Continuous variables were expressed as median and range and compared using the Mann-Whitney *U* test. Categorical variables were expressed as number and percentage and compared using Fisher's exact test. The receiver operating characteristic curve analysis for continuous variables was used to determine the cut-off value. Statistically significant variables from the univariate analysis were entered into the multivariate logistic regression model to identify the independent predictors of complications. P-values less than 0.05 were considered statistically significant.

## 3 Results

A total of 100 patients (65 men, 65.0% and 35 women, 35.0%) with a median age of 68 (range, 20–91) years were enrolled. The median number of hemorrhoid piles was 1 (range, 1–6) and of patients with Goligher classification grades 3 and 4 as 85 (85.0%) and 15 (15.0%), respectively.

The baseline characteristics are shown in Table 1. A significantly greater number of women constituted the elderly group. In the elderly group, the aspartate aminotransferase (AST) levels were significantly higher, albumin levels were lower, cholinesterase (ChE) levels were lower, platelet counts (Plt) were lower, and liver functions were reduced; the blood urea nitrogen (BUN) levels were higher and estimated glomerular filtration rate (eGFR) were significantly lower. The hemoglobin (Hb) levels were significantly lower. There was no difference in the number of hemorrhoid piles or of patients with Goligher classification grades 3 and 4, in the operation time between the elderly and non-elderly group.

**Table 1:** Clinicopathological characteristics of enrolled patients with hemorrhoid.

	Non-elderly, N = 66 (66.0)	Elderly, N = 34 (34.0)	p-value
Age (years)	62 (20–74)	78 (75–91)	<0.001
Sex (Male/Female)	49/17 (74.2/25.8)	16/18 (47.1/52.9)	0.009
BMI (kg/m^2^)	22.9 (17.2–32.2)	25.1 (16.8–29.5)	0.596
Comorbidity	35 (53.0)	21(61.7)	0.524
Hypertension	20 (30.3)	15 (44.1)	0.189
Diabetus mellitus	14 (21.2)	14 (41.2)	0.058
Cardiovascular disease	6 (9.1)	4 (11.7)	0.731
Cerebrovascular disease	3 (4.5)	4 (11.7)	0.224
Chronic renal failure	2 (3.0)	0 (0)	0.546
T. Bil (mg/dL)	0.7 (0.3–2.9)	0.6 (0.3–1.4)	0.892
AST (IU/L)	20 (10–49)	23 (15–30)	0.015
ALT (IU/L)	16 (3–99)	18 (4–36)	0.619
LDH (IU/L)	177 (135–377)	182 (136–251)	0.727
CPK (IU/L)	88 (34–439)	81 (27–241)	0.571
Alb (g/dL)	4.3 (3.3–5)	4.2 (2.8–4.6)	0.044
ChE (IU/L)	348 (192–760)	307 (94–428)	0.001
BUN (mg/dL)	14.7 (6.9–35.1)	15.8 (8.4–42.5)	0.004
Cr (mg/dL)	0.77 (0.42–8.07)	0.85 (0.38–1.66)	0.197
eGFR (ml/min)	77.1 (4.41–111)	60.1 (22.7–118)	<0.001
T-Chol (mg/dL)	204 (131–305)	205 (108–243)	0.574
TG (mg/dL)	111 (52–711)	128 (89–409)	0.084
CRP (mg/dL)	0.05 (0.02–13.8)	0.07 (0.02–4.55)	0.219
WBC (/mL)	6200 (3400–15600)	6250 (3100–10000)	0.951
Hb (g/dL)	14.4 (9.2–17.4)	12.4 (7.3–16.6)	0.005
Plt (x10^4^/mm^3^)	26.9 (15.3–359)	20.5 (7.7–279)	0.008
CAR	0.014 (0.004–3.741)	0.015 (0.004–1.197)	0.236
NLR	2.17 (0.93–7.92)	2.26 (1.41–7.49)	0.475
LMR	3.41 (1.11–7.68)	3.82 (1.53–6.95)	0.359
Goligher classification Grade 3/Grade 4	53/13 (80.3/19.7)	32/2 (94.1/5.9)	0.081
Number of hemorrhoid piles	3 (1–6)	3 (1–4)	0.782
Operation time	38 (7–130)	37 (6–56)	0.366

*Alb* albumin, *AST* asparate aminotransferase, *ALT* alanine aminotransferase, *BMI* body mass index, *BUN* blood urea nitrogen, *CAR* C-reactive protein to albumin ratio, *Cr* creatinine, *CRP* C-reactive protein, *CPK* creatine phosphokinase, *eGFR* estimated glomelular filtration rate, *LDH* lactate dehydrogenase, *LMR* lymphocyte to monocyte ratio, *NLR* neutrophil to lymphocyte ratio, *Plt* platelet count, *T. Bil* total bilirubin, *T. Chol* total cholesterol, *TG* triglyceride, *WBC* white blood cell.

Data were presented as mean and interquartile ranges of continuous variables, and as number and percentage for categorized variables.

Adverse events in the present study are detailed in Table 2. The incidence of postoperative complications was not significantly different between the two groups. One patient experienced postoperative bleeding in the non-elderly group, which was resolved after treatment with suturing. Three patients with anal fissures were treated with ointment and lateral internal sphinctelotomy. Rectal stenosis developed in two patients in the non-elderly group and none of the patients in the elderly group. These patients were successfully managed with operative division of the internal sphincter muscle of anus. Two patients in the elderly group reported persistent pain. After confirming that they had no organic illness, these patients were treated with analgesics as outpatients. Eight patients (12.1%) in the elderly group developed recurrences. The recurrence rate was significantly higher in the elderly group. All these patients underwent reoperation.

**Table 2:** Adverse events.

	Non-elderly, N = 66 (66.0)	Elderly, N = 34 (34.0)	p-value
Total number of adverse events	14 (21.2)	2 (5.9)	0.081
Postoperative bleeding	1 (1.5)	0 (0)	1
Anal fissure	3 (4.5)	0 (0)	0.549
Rectal stenosis	2 (3.0)	0 (0)	0.546
Anal pain	0 (0)	2 (5.9)	0.113
Recurrence	8 (12.1)	0 (0)	0.048

**Fig. 1:**
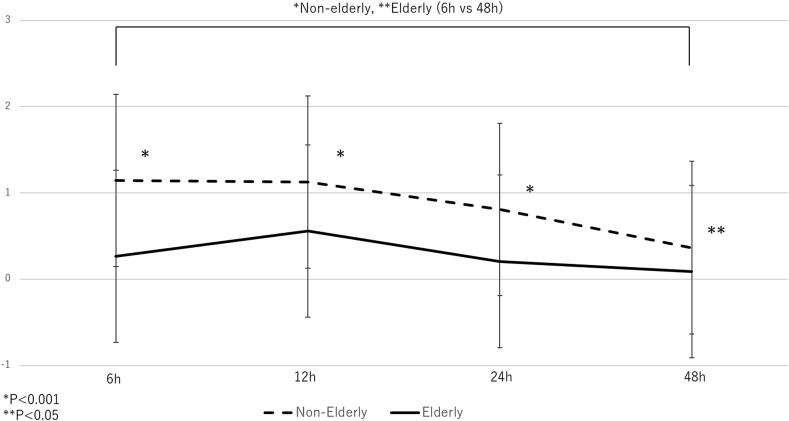
Relationship between the two groups of pain scores by treatment groups and time on study with standard deviation.

The pain score values were measured at 6, 12, 24, and 48 h after surgery. Compared with the values recorded at 6 h postoperatively, the scores tended to decrease at 12 h (P = 0.886) and 24 h (P = 0.127) in the non-elderly group although the difference was not significant. In elderly group, the scores increased at 12 h (p = 0.413) and decreased at 24 h (P = 0.561) compared with the values recorded at 6 h postoperatively, although the difference was not significant. The scores significantly decreased at 48 h after surgery in the non-elderly group and elderly group (p < 0.001 and P = 0.041, respectively). The scores of the elderly group were significantly smaller than those of the non-elderly group. The pain scores at the different time-points are shown in Fig. 1.

The results of the univariate and multivariate analyses for the identification of the risk factors for postoperative complications are summarized in Table 3. Univariate analysis determined male sex, high ALT levels >41 IU/L, high WBC levels >9000/mL, numbers of hemorrhoid piles ≥4, Goligher classification grade 4, operation time >60 min, and NLR >2.15 were statistically significant prognostic factors for complication. The multivariate analysis identified two indicators of complication (Goligher classification grade 4 and high NLR >2.15).

**Table 3:** Univariate and multivariate analysis for risk factor of postoperative complications.

	Univariate analysis			Multivariate analysis		
	OR	95%CI	p-value	OR	95%CI	p-value
Age (years)						
<75						
≥75	4.307	0.918–20.21	0.064			
Sex						
Male	4.529	1.066–21.23	0.049	2.272	0.401–12.87	0.353
Female						
BMI (kg/m2)						
≤22.7						
>22.7	0.471	0.149–1.481	0.197			
Alb (g/dL)						
≥4.0						
<4.0	1.919	0.621–5.942	0.257			
T. Bil (mg/dL)						
≤1.0						
>1.0	0.563	0.116–2.717	0.474			
AST (IU/L)						
≤34						
>34	5.857	0.761–45.05	0.089			
ALT (IU/L)						
≤41						
>41	6.666	1.468–30.26	0.014	7.961	0.972–65.15	0.053
ChE						
≥308						
<308	2.962	0.971–9.049	0.056			
LDH						
≤175						
>175	2.045	0.591–7.071	0.258			
CPK						
≤101						
>101	1.105	0.214–5.697	0.904			
BUN						
≤20.0						
>20.0	0.563	0.116–2.717	0.474			
Cr						
≤1.0						
>1.0	2.777	0.737–10.46	0.131			
eGFR						
≥45						
<45	2.201	0.708–7.111	0.203			
WBC						
≤9000						
>9000	4.601	1.489–14.21	0.008	2.649	0.584–12.01	0.206
Hb						
≤11.9						
>11.9	2.201	0.704–6.868	0.174			
Plt (x10^4^/mm^3^)						
≥35.2						
<35.2	1.776	0.367–8.573	0.474			
CRP (mg/dL)						
≤0.47						
>0.47	0.921	0.178–4.743	0.921			
Number of hemorrhoid piles						
≤3						
≥4	3.601	1.103–11.74	0.033	1.342	0.192–9.371	0.766
Goligher classification						
Grade 3						
Grade 4	5.001	1.467–17.03	0.011	6.005	1.069–33.71	0.029
Operation time (min)						
≤60						
>60	7.801	2.106–28.88	0.002	2.192	0.274–17.49	0.458
CAR						
≤0.123						
>0.123	0.904	0.175–4.663	0.904			
NLR						
≤2.15						
>2.15	3.461	1.032–11.61	0.044	5.172	1.184–22.59	0.028
LMR						
≥4.48						
<4.48	3.499	0.743–16.48	0.113			

*Alb* albumin, *AST* asparate aminotransferase, *ALT* alanine aminotransferase, *BMI* body mass index, *BUN* blood urea nitrogen, *CAR* C-reactive protein to albumin ratio, *Cr* creatinine, *CRP* C-reactive protein, *CPK* creatine phosphokinase, *eGFR* estimated glomelular filtration rate, *LDH* lactate dehydrogenase, *LMR* lymphocyte to monocyte ratio, *NLR* neutrophil to lymphocyte ratio, *Plt* platelet count, *T. Bil* total bilirubin, *T. Chol* total cholesterol, *WBC* white blood cell.

## 4 Discussion

In this study, we aimed to evaluate the safety and efficacy of hemorrhoidectomy for the elderly patients with hemorrhoids and found that the elderly group had higher proportion of women, higher levels of AST, lower levels of albumin, ChE, Plt, (suggesting impairment of liver function), and lower eGFR and hemoglobin levels, (suggesting impairment of kidney function). We found that age itself was not a risk factor for postoperative complications. Among the patients with hemorrhoids in this study, the risk factors for postoperative complications were identified as Goligher classification grade 4 and a high NLR.

Elderly patients with unique challenges, because they have physiologic, pharmacologic, psychologic, and social attributes not present in younger patients [18,19]. The elderly usually have a higher incidence of comorbidities, including hypertension, diabetes, respiratory disease, heart disease, kidney disease, and malnutrition [4]. In the past, these comorbidities in the elderly would increase morbidity and mortality, and they were considered a high-risk group for surgery [20]. Improving the quality of surgical care in the elderly requires careful preoperative evaluation, risk assessment, optimal surgical technique, and anesthetic management [18]. However, in this study, we showed that elderly patients had more comorbidities, but that postoperative complication rates did not differ between the elderly and non-elderly patients. In particular, the incidence of recurrences was significantly low in the elderly group. These results may indicate that screening in surgery patients and improvement of surgical technique and perioperative care would ensure that surgery preserves as much quality of life as possible.

After the operation, the degree of pain in the questionnaire was recorded by the pain score. In this study, postoperative pain was effectively reduced [21]. Pain was significantly reduced, especially in the elderly. The reason for this is that, in addition to pulling and expanding in various directions with a stalk anoscope at the start of surgery, individual excision of main hemorrhoids and ligature of accessory hemorrhoids alone in some cases may be affected. The width of the resection is recommended to be about half the width of the hemorrhoid. The internal anal sphincter of patients with hemorrhoids exhibits abnormal rhythm of contraction and contraction of the anus requires more pressure than a healthy person [22]. Preservation of the sphincter muscle when exfoliating the hemorrhoidal tissue is associated with postoperative pain relief due to the abnormality of the sphincter is associated with hemorrhoid. Moreover, Fig. 1 shows that pain is properly managed by administration on the clinical path, which helps prevent decline for activity of daily life din elderly patients.

Multivariate analyses indicated that Goligher classification Grade 4 and high NLR levels as independent risk factors for the development of complications in patients with hemorrhoids. This applies to Grade IV hemorrhoids, as previously reported [23], and is known to increase the incidence of complications. It is necessary to prevent stenosis from a radial incision due to a circumferential incision at the time of an emergency operation in an incarcerated state, or to prevent recurrence due to a leftover. On the other hand, recently, it was found that NLR was associated with severity and poor prognosis of many inflammatory diseases [[24], [25], [26]]. Neutrophils play an important role in regulating the inflammatory response, and neutrophilia is considered a novel inflammatory marker [25]. Lymphopenia is often secondary to increased corticosteroid levels in acute stress conditions [27]. It is necessary to pay attention to the risk of complications in patients hemorrhoids with strong inflammation.

This study has several limitations including the retrospective nature and the relatively small sample size. Not all elderly patients with hemorrhoids were not evaluated in this study. There may have been selection bias regarding cases determined to be inoperable by preoperative evaluation were not included, but we imposed strict inclusion and exclusion criteria to mitigate this bias. The small number of cases did not allow us to identify risk factors for complications in the elderly alone. We had a small sample size and yet we were able to achieve statistically significant results of the risk factors in patients with hemorrhoids.

In conclusion, age itself is not an independent negative prognostic factor for postoperative complications in patients with hemorrhoids. Among the patients with hemorrhoids in this study, the risk factors for postoperative complications were Goligher classification grade 4 and high NLR. These findings suggest that, in the absence of these conditions, hemorrhoidectomy may be performed in patients with hemorrhoids, irrespective of the age.

## Funding

There is no grant support or financial relationship.

## Author contribution

Masateru Yamamoto, Masanobu Ikeda, and Hideki Ohdan were involved in the study concept and design, data acquisition and analysis, drafting of the manuscript. Tomio Matsumoto, Masahiko Takemoto, Ryo Sumimoto, and Tsuyoshi Kobayashi contributed to perform data analysis and interpretation and to draft the manuscript.

## Declaration of competing interest

All author declared no conflicts of interest.
